# Effect of Empagliflozin Treatment on Ventricular Repolarization Parameters

**DOI:** 10.31083/j.rcm2502064

**Published:** 2024-02-18

**Authors:** Fatih Ozturk, Hasim Tuner, Adem Atici, Hasan Ali Barman

**Affiliations:** ^1^Department of Cardiology, Yuzuncu yil University, Faculty of Medicine, 65080 Van, Turkey; ^2^Department of Cardiology, Istanbul Medeniyet University, Faculty of Medicine, Goztepe Training and Research Hospital, 34722 Istanbul, Turkey; ^3^Department of Cardiology, Istanbul University – Cerrahpaşa, Institute of Cardiology, 34390 Istanbul,Turkey

**Keywords:** empagliflozin, type 2 diabetes mellitus, ventricular repolarization, QT interval, arrhythmias, SGLT-2 inhibitor

## Abstract

**Background:**

An example of a sodium-glucose cotransporter-2 (SGLT-2) inhibitor is Empagliflozin. It is a new medicine for treating type 2 diabetes mellitus (T2DM), but there is increasing interest in how empagliflozin affects the heart. This study aims to examine the impact of empagliflozin treatment on ventricular repolarization parameters in T2DM patients.

**Methods:**

T2DM patients were included in a prospective study. Measurements of ventricular repolarization parameters, including QT interval, corrected QT interval (QTc), QT dispersion (QTd), Tpeak-to-Tend interval (Tp-e), and Tpeak-to-Tend interval corrected for QTc (Tp-e/QTc), were obtained before initiating empagliflozin treatment and six months following treatment initiation. Statistical analysis was performed to assess changes in these parameters.

**Results:**

In this study, 95 patients were diagnosed with T2DM out of 177 patients. Among T2DM patients, 40 were male (42%) compared to 48% males in controls (*p* = 0.152). The average age of the T2DM patients was 60.2 ± 9.0 years, compared to 58.2 ± 9.2 years in the control group (*p* = 0.374). When comparing pre- and post-treatment measurements of parameters representing ventricular repolarization (QT 408.5 ± 22.9/378.8 ± 14.1, *p* < 0.001; QTc 427.0 ± 20.5/404.7 ± 13.8, *p* < 0.001; QTd 52.1 ± 1.2/47.8 ± 1.7, *p* < 0.001; Tp-e 82.3 ± 8.7/67.1 ± 5.1, *p* < 0.001; Tp-e/QTc 0.19 ± 0.01/0.17 ± 0.01, *p* < 0.001 (respectively)), statistically significant improvements were observed. A statistically significant dose-dependent decline in the magnitude of change in the QTc parameter (19.4/29.6, *p* = 0.038) was also observed.

**Conclusions:**

According to these results, empagliflozin may decrease the risk of potential ventricular arrhythmias.

## 1. Introduction

There has been a consistent increase in the global occurrence of type 2 diabetes mellitus (T2DM). It is predicted that there will be 500 million people with diabetes by 2035, and that one in three people could have T2DM by 2050 [1, 2]. T2DM is a significant cause of cardiovascular disease [3]. T2DM also damages the heart’s conduction system, leading to cardiac neuropathy, which affects around a third of people with T2DM [1]. This damage can increase sympathetic nervous system activity, potentially affecting cardiac repolarization and causing fatal ventricular arrhythmias, independent of disorders like hypertension, coronary artery disease (CAD), or heart failure [2].

Metabolic and mechanical abnormalities in CAD can lead to a greater degree of repolarization dispersion, which in some cases may be the basis for ventricular arrhythmias. The QT interval, as seen in electrocardiography, represents the repolarization of the heart. Numerous studies have shown that the QT interval represents potentially fatal ventricular arrhythmias [4, 5]. In addition to the QT interval, changes in the QT dispersion and T wave are also used to assess arrhythmias [6]. Research suggests that the difference between the highest and lowest points of the T wave (Tp-e) on the electrocardiogram (ECG) is more sensitive than the QT interval in predicting fatal ventricular arrhythmias [7, 8, 9]. In addition, it has been determined that the Tp-e/QT ratio is an important indicator of ventricular arrhythmias [6].

Compelling evidence has been offered in recent studies regarding the efficacy of sodium-glucose co-transporter 2 (SGLT-2) inhibitors in preventing cardiovascular complications [10, 11, 12]. In particular, the EMPA-REG (Empagliflozin Cardiovascular Outcome Event Trial in Type 2 Diabetes Mellitus Patients-Removing Excess Glucose) study demonstrated how empagliflozin was effective in reducing hospitalizations related to both glycemic control as well as heart failure in people with T2DM [13]. SGLT2 inhibitors significantly reduce mortality, major adverse cardiac events, nonfatal heart attacks and heart failure in T2DM patients, according to a meta-analysis. Interestingly, different SGLT-2 inhibitor subtypes appear similarly beneficial [11]. However, the effect of SGLT-2 inhibitors on cardiac electrical activity and the risk of ventricular arrhythmias is unclear.

The objective of this study is to investigate the way empagliflozin affects ECG parameters in T2DM patients associated with ventricular arrhythmias.

## 2. Materials and Methods

### 2.1 Study Population

**Fig. 1.**
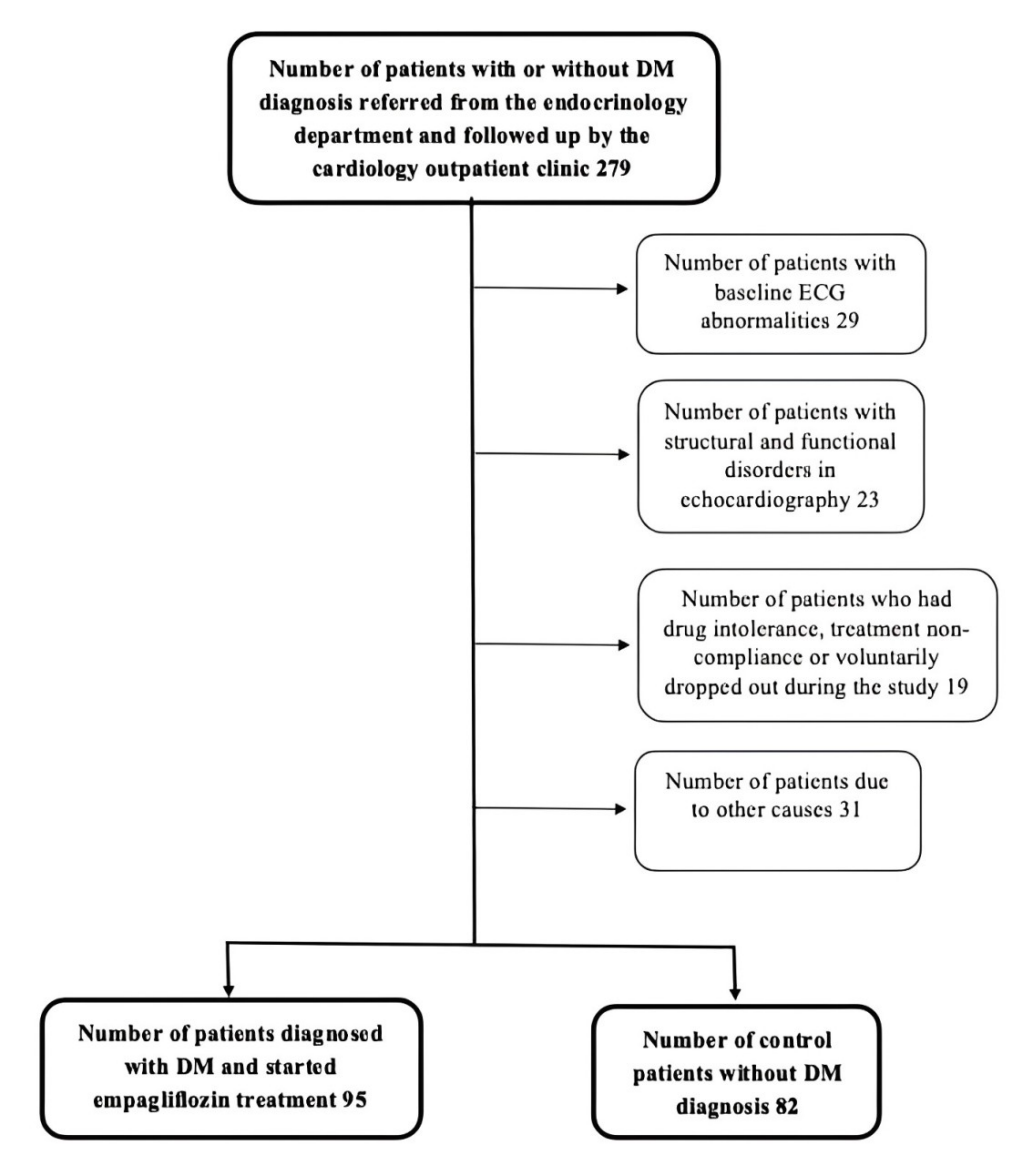
**Flow chart of the study design**. DM, diabetes mellitus; ECG, electrocardiogram.

This is an observational, prospective, single-centre research. Patient enrolment began in January 2021, and each patient was followed up after six months. In total, the study included 177 patients, 95 with T2DM and 82 controls. T2DM patients who had been started on the SGLT-2 inhibitor empagliflozin by the internal medicine and endocrinology departments at our hospital, as well as those without a history of T2DM, were included in the study. Patients with hemoglobin A1c (HbA1c) levels persistently above 6.5% despite treatment were prescribed empagliflozin at doses of either 10 mg or 20 mg. Forty-five patients not meeting the inclusion criteria were excluded (Fig. 1). Exclusion criteria included a history of type 1 diabetes or other specific types of diabetes, heart failure (including that with preserved ejection fraction), modest to intense valvular heart disease, bundle branch block or atrioventricular block on ECG, atrial fibrillation, permanent cardiac pacing, history of pulmonary embolism, pulmonary hypertension, malignancy, familial hyperlipidemia, intolerance to chronic kidney disease medications and pregnancy. Those patients that required switching medication (including antidiabetic, antiarrhythmic, antihypertensive and antihyperlipidemic drug classes) were also eliminated to offset potential metabolic effects of non-SGLT-2 inhibitors, or if they had unstable medical conditions (such as decompensated heart failure, acute coronary syndrome, or unstable arrhythmias) during the study period.

Biochemical analyses, complete blood counts and HbA1c measurements were performed prior to treatment. For every patient, body mass index (BMI) was calculated. A total of twelve-lead surface ECGs were carried out and patients underwent echocardiography before starting empagliflozin treatment. At the end of the sixth month, all these assessments were repeated. The Declaration of Helsinki was adopted by the study protocol, which was accepted by the local medical ethics committee. Each subject had the study protocol explained to them in detail, after which written informed consent was obtained.

### 2.2 Transthoracic Echocardiography

Echocardiographic evaluations, including M-mode, two-dimensional, and Doppler echocardiography, were conducted using the Vivid E9 instrument and the X5-1 transthoracic probe (Vivid 9 Pro, General Electric Medical Systems, Milwaukee, WI, USA). These assessments were performed in the left lateral position following a rest period of at least 15 minutes. Echocardiography was carried out in accordance with the standard images and techniques outlined in the directives of the American Society of Echocardiography (ASE) [14].

### 2.3 Electrocardiography

For every patient, a 12-lead ECG was noted following a 30-minute rest period in a room at 20–24 °C. Participants were required to fast for at least 2 hours, abstain from smoking and alcohol consumption for the previous 24 hours, and avoid strenuous physical activity prior to ECG recording. All ECGs were performed with the following settings: filter range between 0.5 Hz and 150 Hz, AC filter of 60 Hz, paper speed of 25 mm/s and amplitude of 10 mm/mV. All standard 12-lead ECGs were examined independently by two clinicians blinded to the study design and clinical data. On-screen digital caliper software (Cardio Calipers version 3.3, Iconico, Inc., New York, NY, USA) was used to digitize ECG data and for each lead, the mean value for the three measurements was computed. The QT interval was determined from the onset of the QRS complex till the T wave ended, and the Bazett formula (QTc = QT√[R-R interval]) was used to calculate the corrected QT interval (QTc) from the heart rate. QT dispersion (QTd) was calculated as the variation between the maximum and minimum QT intervals over all 12 leads. The time from when the T wave peaked till it reached its end was represented by the Tp-e interval that was measured from the precordial leads [15].

**Fig. 2.**
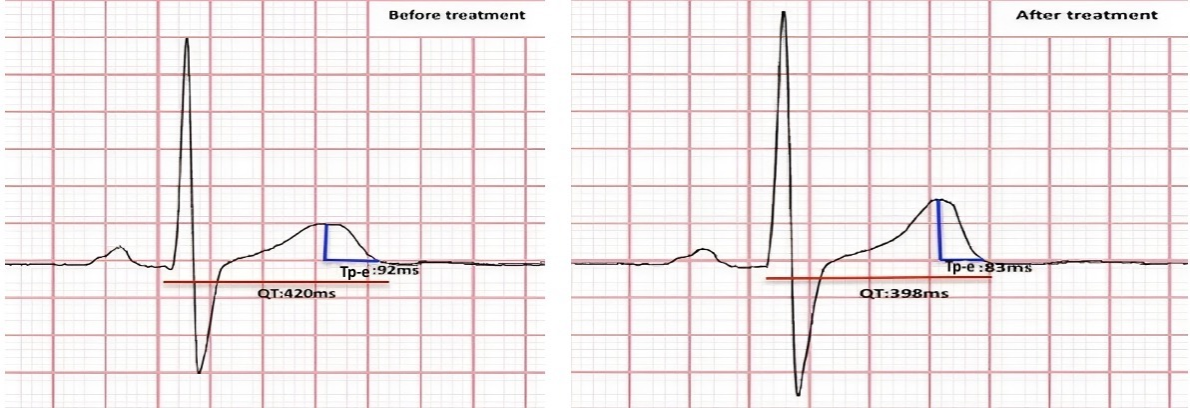
**Changes in the pre-treatment and post-treatment Tp-e and corrected QT (QTc) values on ECG**. ECG, electrocardiogram; Tp-e, Tpeak-to-Tend interval; QTc, corrected QT interval.

Six months after starting treatment with empagliflozin, patients’ QT, QTc, QTd and Tp-e values were re-evaluated and compared with their baseline values (Fig. 2).

### 2.4 Statistical Analysis

SPSS 25.0 for Windows (SPSS Inc., Chicago, IL, USA) was used to perform all statistical tests. The normality of the data was analyzed by employing the Kolmogorov-Smirnov test. To represent normally distributed numerical data, mean ± SD was used, while median (25–75) percentiles were used to represent non-normally distributed parameters. Furthermore, percentages were used to represent categorical data. To perform a comparison between unpaired samples, the student’s *t*-test or Mann-Whitney U test was employed, when appropriate. To examine changes in parameters observed before and after treatment with empagliflozin, a paired two-sample *t*-test was employed for normally distributed data, while non-normally distributed data was tested using the Wilcoxon test. Pearson’s rank correlation test was used to analyze the relationships between variables. To examine which independent variables influenced the dependent variable QTc, multiple linear regression analyses were performed by utilizing the stepwise technique. Multicollinearity was tested for all independent variables in the multiple linear regression. Variables were considered collinear when the variance inflation factor (VIF) was greater than 3.0. All reported confidence intervals (CIs) are calculated at the 95% level of confidence. Reproducibility was assessed by re-analysis of 20 randomly selected patients and reported as intra-observer reliability. Inter-observer reproducibility was calculated by a second independent observer. Significance was considered to be at a 2-sided *p* < 0.05.

## 3. Results

Of the 177 patients who were part of the study, 95 were diagnosed with T2DM and their clinical and demographic attributes are depicted in Table 1. Of the 95 T2DM patients, 40 were male (42%), whereas in the control group of 82 patients, 40 were male (48%) (*p* = 0.152). The average age of the T2DM patients was 60.2 ± 9.0 years, while the average age of the control group was 58.2 ± 9.2 years (*p* = 0.374). No statistically significant differences existed between the groups with regard to BMI, systolic arterial pressure (SAP), diastolic arterial pressure diastolic arterial pressure (DAP), smoking status, hypertension (HT), hyperlipidemia (HPL) and incidence of CAD. No significant differences were observed between the two groups in terms of hemogram, white blood cell count, creatinine, sodium, potassium, calcium and magnesium. As expected, there were significantly greater levels of HbA1c in the T2DM group. In addition, when comparing the medical treatments received by the patients between the groups, the use of angiotensin-converting enzyme inhibitors/angiotensin receptor blockers (ACE-I/ARB), calcium channel blockers (CCB) and beta-blockers (BB) was similar, whereas the use of acetylsalicylic acid (ASA) and statins was considerably greater in the T2DM group. Finally, when the doses of empagliflozin treatment were determined, it was found that in the T2DM group, a dose of 10 mg was given to 68 patients (72%) and a dose of 20 mg was given to 27 patients (28%).

**Table 1.** **Comparison of the characteristics of individuals with and without a history of diabetes mellitus**.

Characteristic		DM patients (n = 95)	Control (n = 82)	*p*
Age (years)		60.2 ± 9.0	58.2 ± 9.2	0.152
Male, n (%)		40 (42%)	40 (48%)	0.374
BMI (${\mathrm{kg/m}}^{2}$)		26.2 ± 4.8	24.9 ± 4.6	0.067
Smoking, n (%)		55 (58%)	42 (51%)	0.374
SAP (mmHg)		121.3 ± 15.6	118.5 ± 15.9	0.237
DAP (mmHg)		62.3 ± 11.3	65.0 ± 9.4	0.091
Chronic medical illness				
	HT, n (%)	57 (60%)	44 (53%)	0.395
	DM, n (%)	95 (100%)	-	-
	HPL, n (%)	41 (43%)	31 (37%)	0.470
	CAD, n (%)	43 (45%)	27 (33%)	0.094
Laboratory findings				
	Haemoglobin (g/dL)	13.1 ± 1.2	12.9 ± 1.0	0.118
	WBC (${\mathrm{10}}^{3}$/µL)	7.0 ± 1.8	7.2 ± 1.3	0.273
	Creatinine (mg/dL)	0.95 ± 0.12	0.92 ± 0.11	0.080
	Sodium (mmol/L)	139.5 ± 4.6	138.7 ± 3.9	0.530
	Potassium (mmol/L)	4.1 ± 0.3	4.2 ± 0.9	0.203
	Calcium (mg/dL)	9.3 ± 0.8	9.2 ± 0.7	0.850
	Magnesium (mg/dL)	1.9 ± 0.1	2.0 ± 0.2	0.166
	HbA1c (%)	9.1 ± 1.4	5.0 ± 0.3	<0.001
Treatment				
	ACE-I/ARB, n (%)	67 (70%)	49 (59%)	0.133
	BB, n (%)	71 (74%)	50 (61%)	0.051
	CCB, n (%)	20 (21%)	27 (32%)	0.074
	ASA, n (%)	61 (64%)	32 (39%)	0.001
	Statin, n (%)	50 (52%)	27 (33%)	0.008
Empagliflozin, n (%)				
	10 mg start	68 (72%)	-	-
	20 mg start	27 (28%)	-	-

Abbreviations: BMI, body mass index; SAP, systolic arterial pressure; DAP, diastolic arterial pressure; HbA1c, hemoglobin A1c; HT, hypertension; DM, diabetes mellitus; HPL, hyperlipidemia; CAD, coronary artery disease; ACE-I, angiotensin converting enzyme inhibitor; ARB, angiotensin receptor blocker; BB, beta-blockers; CCB, calcium channel blocker; ASA, acetylsalicylic acid; WBC, white blood cell.

Table 2 demonstrates the conventional echocardiographic and electrocardiographic properties of the patients taking part in the study. In the conventional echocardiographic assessment, the left ventricular ejection fraction (LVEF) was statistically significantly less in the T2DM patients in comparison to the control group (LVEF 58.6 ± 8.0/60.9 ± 5.7, *p* = 0.029). While there was no between-group difference in left ventricular end-diastolic dimension (LVEDD), in the empagliflozin group with T2DM (LVESD 30.7 ± 2.2/28.8 ± 2.9, *p* < 0.001), left ventricular end-systolic dimension (LVESD) was considerably higher. Similarly, no significant variations existed between groups in left ventricular wall thickness and left atrial diameter. Regarding the parameters of left ventricular diastolic function, E wave, A wave and E/A ratio did not show statistically significant variations among the groups. When comparing the electrocardiographic properties among the groups, the heart rate (HR) did not differ, but the baseline values of QT, QTc, QTd, Tp-e and Tp-e/QTc were statistically significantly larger in the group of patients with T2DM (QT 408. 5 ± 22.9/370.9 ± 20.2, *p* < 0.001; QTc 427.0 ± 20.5/385.8 ± 16.0, *p* < 0.001; QTd 52.1 ± 1.2/46.2 ± 2.1, *p* < 0.001; Tp-e 82.3 ± 8.7/61.2 ± 5.8, *p* < 0.001; Tp-e/QTc 0.19 ± 0.01/0.16 ± 0.01, *p* < 0.001).

**Table 2.** **Comparison of conventional echocardiographic and electrocardiographic features of individuals with and without diabetes mellitus**.

Conventional echocardiographic findings on admission hospital			
	T2DM patients (n = 95)	Control (n = 82)	
LVEF (%)	58.6 ± 8.0	60.9 ± 5.7	0.029
LVEDD (mm)	47.2 ± 4.4	46.2 ± 3.6	0.103
LVESD (mm)	30.7 ± 2.2	28.8 ± 2.9	<0.001
IVS (mm)	9.6 ± 1.5	9.6 ± 1.3	0.981
PW (mm)	9.6 ± 1.4	9.4 ± 1.3	0.346
LA (mm)	32.4 ± 4.7	32.6 ± 3.6	0.842
E (cm/s)	76.0 ± 14.9	73.8 ± 17.3	0.377
A (cm/s)	68.2 ± 14.3	65.6 ± 15.3	0.244
E/A ratio	1.1 ± 0.3	1.1 ± 0.3	0.833
Electrocardiography findings on admission hospital			
HR (beat/min)	71.8 ± 11.1	74.5 ± 9.7	0.093
QT (msec)	408.5 ± 22.9	370.9 ± 20.2	<0.001
QTc (msec)	427.0 ± 20.5	385.8 ± 16.0	<0.001
QTd (msec)	52.1 ± 1.2	46.2 ± 2.1	<0.001
Tp-e (msec)	82.3 ± 8.7	61.2 ± 5.8	<0.001
Tp-e/QTc ratio	0.19 ± 0.01	0.16 ± 0.01	<0.001

Abbreviations: LVEF, left ventricle systolic diameter; LVEDD, left ventricle end-diastolic diameter; LVESD, left ventricle end-sistolic diameter; IVS, interventicular septum; PW, posterior wall; LA, left atrium; HR, heart rate; QTc, corrected QT interval; QTd, QT dispersion; Tp-e, Tpeak-to-Tend interval; T2DM, type 2 diabetes mellitus.

The blood pressure and laboratory results of the patient group before and following treatment are summarised in Table 3. There was no significant variation in blood pressure before and following treatment. Although no significant difference was observed in haemogram, white blood cell count, creatinine, sodium, potassium, calcium and magnesium results, a significant difference was noted in the levels of HbA1c (HbA1c 9.1 ± 1.4/8.0 ± 1.0; *p* < 0.001).

**Table 3.** **Comparison of blood pressure and laboratory findings before and after empagliflozin treatment**.

Parameters	Before the treatment	After the treatment	*p*
SAP (mmHg)	121.3 ± 15.6	117.1 ± 17.2	0.095
DAP (mmHg)	62.3 ± 11.3	61.9 ± 10.9	0.179
Haemoglobin (g/dL)	13.1 ± 1.2	13.0 ± 1.1	0.085
WBC (${\mathrm{10}}^{3}$/µL)	7.0 ± 1.8	7.1 ± 1.8	0.135
Creatinine (mg/dL)	0.958 ± 0.125	0.958 ± 0.120	0.058
Sodium (mmol/L)	139.5 ± 4.6	139.2 ± 4.4	0.021
Potassium (mmol/L)	4.13 ± 0.33	4.14 ± 0.33	0.051
Calcium (mg/dL)	9.3 ± 0.8	9.2 ± 0.7	0.034
Magnesium (mg/dL)	1.97 ± 0.15	1.96 ± 0.14	0.158
HbA1c (%)	9.1 ± 1.4	8.0 ± 1.0	<0.001

Abbreviations: SAP, systolic arterial pressure; DAP, diastolic arterial pressure; WBC, white blood cell; HbA1c, hemoglobin A1c.

**Fig. 3.**
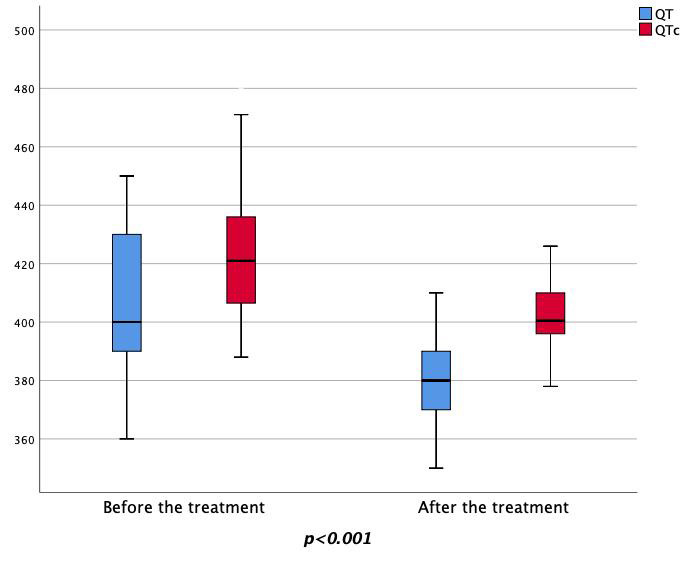
**Changes in the pre-treatment and post-treatment QT and QTc values**. QTc, corrected QT interval.

**Fig. 4.**
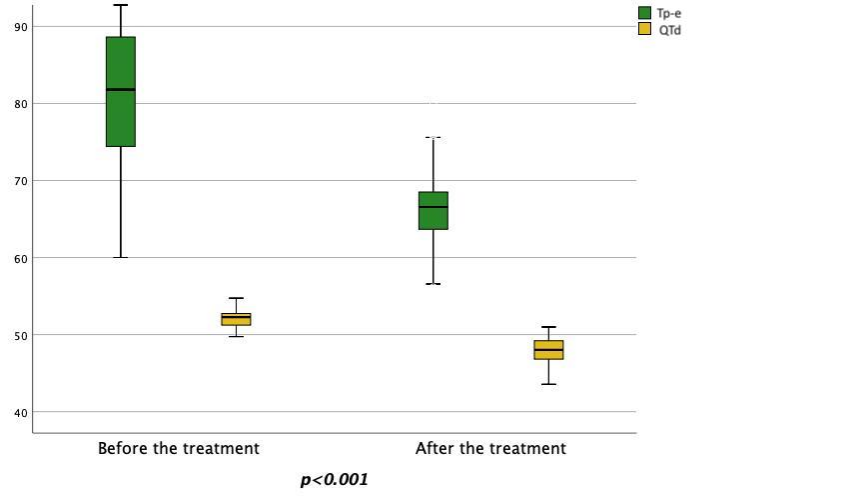
**Changes in the pre-treatment and post-treatment Tp-e and QTd values**. Tp-e, Tpeak-to-Tend interval; QTd, QT dispersion.

**Fig. 5.**
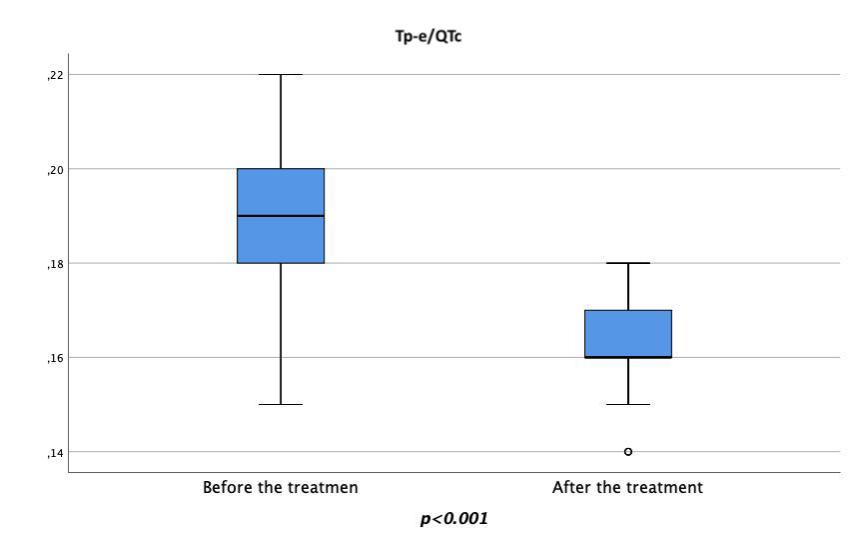
**Changes in the pre-treatment and post-treatment Tp-e/QTc ratio. **Tp-e, Tpeak-to-Tend interval; QTc, corrected QT interval.

Table 4 depicts the conventional echocardiographic and electrocardiographic characteristics of patients with T2DM prior to and following empagliflozin treatment. Regarding echocardiographic parameters, there were no significant changes in LVEF, LVEDD, LVESD, IVS, PW, LA, E wave, A wave and E/A ratio values between the pre- and post-treatment assessments. However, when examining the electrocardiographic parameters, no differences in heart rate (HR) were observed between the pre-treatment and post-treatment phases. In particular, the values of QT, QTc, QTd, Tp-e and Tp-e/QTc showed statistically significant reductions after empagliflozin treatment compared to pre-treatment values (QT 408.5 ± 22.9/378.8 ± 14. 1, *p* < 0.001; QTc 427.0 ± 20.5/404.7 ± 13.8, *p* < 0.001; QTd 52.1 ± 1.2/47.8 ± 1.7, *p* < 0.001; Tp-e 82.3 ± 8.7/67.1 ± 5.1, *p* < 0.001; Tp-e/QTc 0.19 ± 0.01/0.17 ± 0.01, *p* < 0.001) (Figs. 3,4,5).

**Table 4.** **Comparison of conventional echocardiographic and electrocardiographic features before and after empagliflozin treatment**.

Echocardiographic parameters	Before the treatment	After the treatment	*p*
LVEF (%)	58.6 ± 8.0	59.8 ± 8.5	0.254
LVEDD (mm)	47.2 ± 4.4	46.6 ± 3.6	0.287
LVESD (mm)	30.7 ± 2.2	30.1 ± 2.9	0.139
IVS (mm)	9.6 ± 1.5	9.6 ± 1.3	0.916
PW (mm)	9.6 ± 1.4	9.5 ± 1.4	0.683
LA (mm)	32.4 ± 4.7	32.3 ± 3.5	0.080
E (cm/s)	76.0 ± 14.9	72.6 ± 14.3	0.105
A (cm/s)	68.2 ± 14.3	66.9 ± 14.6	0.503
E/A ratio	1.1 ± 0.3	1.0 ± 0.4	0.097
Electrocardiography parameters			
HR (beat/min)	71.8 ± 11.1	74.2 ± 10.6	0.174
QT (msec)	408.5 ± 22.9	378.8 ± 14.1	<0.001
QTc (msec)	427.0 ± 20.5	404.7 ± 13.8	<0.001
QTd (msec)	52.1 ± 1.2	47.8 ± 1.7	<0.001
Tp-e (msec)	82.3 ± 8.7	67.1 ± 5.1	<0.001
Tp-e/QTc ratio	0.19 ± 0.01	0.17 ± 0.01	<0.001

Abbreviations: LVEF, left ventricle systolic diameter; LVEDD, left ventricle end-diastolic diameter; LVESD, left ventricle end-sistolic diameter; IVS, interventicular septum; PW, posterior wall; LA, left atrium; HR, heart rate; QTc, corrected QT interval; QTd, QT dispersion; Tp-e, Tpeak-to-Tend interval.

The mean difference values for conventional echocardiographic and electrocardiographic characteristics before and after empagliflozin treatment, grouped by treatment dose in patients with T2DM, are shown in Table 5. In this analysis, no significant differences in LVEF, LVEDD, LVESD and E/A wave values were observed between treatment doses. However, in the group receiving the 20 mg dose of empagliflozin, changes in left atrium (LA) values were increasingly prominent, showing a statistically significant difference (Delta-LA –0.02/0.55, *p* = 0.026). When electrocardiographic parameters were similarly examined, changes in HR, QT, QTd, Tp-e and Tp-e/QTc were not influenced by empagliflozin dose. However, the QTc showed a more pronounced decrease in the 20 mg empagliflozin group, with a statistically significant reduction (QTc 19.4/29.6, *p* = 0.038).

**Table 5.** **Comparison of Empagliflozin treatment dose with conventional echocardiographic and electrocardiographic features**.

Echocardiographic parameters	Empagliflozin 10 mg (n = 68)	Empagliflozin 20 mg (n = 27)	*p*
	Mean difference	Mean difference	
Delta-LVEF (%)	–1.75	0.14	0.420
Delta-LVEDD (mm)	0.52	0.81	0.823
Delta-LVESD (mm)	1.08	–0.55	0.075
Delta-LA (mm)	–0.02	0.55	0.026
Delta-E/A ratio	0.07	0.16	0.402
Electrocardiography parameters			
HR (beat/min)	–0.64	–6.62	0.115
QT (msec)	31.9	24.0	0.135
QTc (msec)	19.4	29.6	0.038
QTd (msec)	4.43	3.65	0.067
Tp-e (msec)	14.1	17.5	0.055
Tp-e/QTc ratio	0.02	0.02	0.317

Abbreviations: LVEF, left ventricle systolic diameter; LVEDD, left ventricle end-diastolic diameter; LVESD, left ventricle end-sistolic diameter; LA, left atrium; HR, heart rate; QTc, corrected QT interval; QTd, QT dispersion; Tp-e, Tpeak-to-Tend interval.

A stepwise method of linear regression assessment was used to assess the parameters affecting post-treatment QTc values, as shown in Table 6. First, statistically significant parameters and those likely to influence the QTc parameter were included in the model. In this context, the relationship between HbA1c, empagliflozin dose, beta-blocker (BB) use, age, BMI, LVEF, sex, CAD, SAP, Haemoglobin, Creatinine, Potassium, Calcium, Magnesium, ACE-I/ARB use, HT and smoking status with post-treatment QTc was evaluated. HbA1c and empagliflozin dose were identified as independent predictors of post-treatment QTc.

**Table 6.** **Independent factors affecting after empagliflozin treatment QTc in DM patients in stepwise multiple linear regression analysis**.

** ${\mathrm{Coefficients}}^{a}$ **				
Model	Unstandardized coefficients		Standardized coefficients	*p* value
	B	Std. Error	Beta	
(Constant)	263.277	8.615		<0.001
HbA1c	3.836	1.000	0.399	<0.001
Empagliflozin dose	–7.991	3.217	–0.252	0.015
**Excluded ${\mathrm{Variables}}^{a}$**				
Model	B	Partial correlation	Collinearity statistics	*p* value
			Tolerance	
BB	0.072	0.076	0.978	0.467
Age	0.138	0.134	0.816	0.198
BMI	–0.054	–0.058	0.989	0.579
LVEF	0.116	0.118	0.901	0.258
Gender	0.070	0.074	0.952	0.480
CAD	–0.038	–0.041	0.998	0.696
ACE-I/ARB	–0.101	–0.107	0.971	0.305
HT	0.072	0.076	0.971	0.464
Smoke	0.024	0.026	0.989	0.805
SAP	0.063	0.067	0.994	0.552
Haemoglobin	–0.112	–0.119	0.987	0.252
Creatinine	–0.107	–0.114	0.998	0.273
Potassium	0.100	0.104	0.943	0.320
Calcium	0.120	0.128	0.999	0.218
Magnesium	0.041	0.044	0.998	0.675

^a^ Dependent Variable: After empagliflozin treatment QTc. Abbreviations: HbA1c, hemoglobin A1C; BB, beta-blockers; BMI, body mass index; LVEF, left ventricle systolic diameter; CAD, coronary artery disease; ACE-I, angiotensin converting enzyme inhibitor; ARB, angiotensin receptor blocker; HT, hypertension; SAP, systolic arterial pressure; QTc, corrected QT interval; DM, diabetes mellitus.

### Reproducibility

The agreement of echocardiographic and electrocardiographic parameters for intra-observer and inter-observer variability was evaluated in a randomly selected group of 20 patients. The intraclass correlation coefficient for intraobserver and interobserver variability was 0.91 (95% CI, 0.85–0.96) and 0.89 (95% CI, 0.81–0.95) for echocardiographic measurements and 0.92 (95% CI, 0.86–0.96) and 0.90 (95% CI, 0.84–0.96) for electrocardiographic measurements.

## 4. Discussion

We investigated the impact of empagliflozin on ECG ventricular repolarisation factors in patients with T2DM in this research. Our findings can be summarised as follows: (i) We observed that the QT, QTc, QTd, Tp-e and Tp-e/QTc values significantly varied among the patient group and the control group. (ii) We observed a significant variation in QT, QTc, QTd, Tp-e and Tp-e/QTc values within the patient group when comparing measurements taken before empagliflozin treatment and those obtained six months after the start of treatment. (iii) We analysed the difference in ventricular repolarisation factors on the ECG based on the treatment dose and found a statistically significant dose-related reduction in the QTc parameter. (iv) Using linear regression analysis, we identified HbA1c levels and empagliflozin treatment dose as independent factors affecting QTc.

Empagliflozin, a potent SGLT-2 inhibitor, is the first glucose-lowering medicine shown to reduce cardiovascular events [16]. Despite several studies suggesting potential effects on atherosclerosis, cardiac remodelling or inflammatory processes, the precise pathophysiology underlying its cardiac effects remains unclear. There is consensus that the drug acts through a complex mechanism of action [17, 18, 19].

The effect of this drug on heart rhythm is also unclear. Some studies have shown that therapeutic doses of the drug do not prolong the QT interval [20], others have reported longer QT intervals in people with T2DM and CAD in comparison to the general population [18, 20]. We also noticed in our research that ventricular repolarisation parameters (QT, QTc, QTd, Tp-e and Tp-e/QTc) were all considerably prolonged in the patient group in contrast to the control group. However, at the six-month follow-up after the addition of empagliflozin to glucose control, a significant shortening of these ventricular repolarisation parameters (QT, QTc, QTd, Tp-e and Tp-e/QTc) was observed.

Although QTd has historically been considered a marker of increased repolarization dispersion and a useful indicator of arrhythmogenic events, contemporary preferences have shifted to more sensitive parameters for predicting arrhythmogenic events on ECGs [21]. Nonetheless, several studies have pointed to impaired QT intervals and QTd in individuals with T2DM and CAD [22]. It is worth noting that increased QT and QTd values in the presence of autonomic neuropathy and arrhythmias are significant contributors to the possibility of death due to sudden cardiac arrest [8, 23]. In our study, we also observed that in comparison to the control group, both QT and QTd were substantially greater in the patient group and that QTd decreased in the patient group after treatment with empagliflozin. These findings suggest that empagliflozin may be linked to decreasing the possibility of death due to sudden cardiac arrest.

The Tp-e interval and the Tp-e/QT ratio are key markers of ventricular repolarisation and greater dispersion [24]. The findings of recent studies have emphasised that the Tp-e/QT ratio provides a more precise assessment of ventricular repolarisation dispersion compared to QT dispersion, QTc dispersion and Tp-e intervals, independent of heart rate variations. In addition, an elevated Tp-e/QT ratio has been linked to a greater risk of arrhythmogenic events [7, 8, 25]. The primary mechanism of Tp-e interval prolongation is the disruption of ion transfer during ventricular repolarization [26]. The Tp-e interval reflects epicardial and myocardial repolarisation, but subendothelial M-cells are vulnerable to early depolarisation. This vulnerability can lead to arrhythmias, and inappropriate stimulation of M-cells can result in lethal cardiac rhythms such as ventricular fibrillation or ventricular tachycardia [8, 27, 28, 29]. A substantial difference in Tp-e interval and Tp-e/QTc ratio among the patient and control groups was observed in this research. When these parameters were remeasured six months after the start of treatment with empagliflozin, we observed significant improvements compared with baseline values. Therefore, it is reasonable to conclude that empagliflozin may reduce the risk of arrhythmias by decreasing the Tp-e interval as well as the Tp-e/QTc ratio. According to the information available, our study seems to be one of the first to examine the link between empagliflozin and the Tp-e interval, as we could not identify any previous studies investigating this aspect.

In the study carried out by Taha *et al*. [30], a significant association was found between left ventricular diastolic dysfunction (LVDD) and the length of QTc duration. In our study, we found that the values of E and A waves decreased with treatment, but this change was not statistically significant and in this context, we concluded that LVDD does not independently show a significant effect on QTc duration. Of course, we are aware that the hypothesis of our study is not relevant to this issue and in this context, the number of LVDD patients in the patient group is not sufficient to reach such a conclusion [30].

Studies investigating the relationship between empagliflozin dose and QTc in the literature are limited and there is no comprehensive study with long-term results. A study by Ring *et al*. [20] investigated empagliflozin doses of 25 and 200 mg, but found no significant relationship with QTc. It is worth noting that this study was based on data from a 24-hour monitoring period [20]. Treatment of chronic diseases often requires longer-term studies, and our study with a 6-month follow-up suggests that empagliflozin dosing leads to significant changes in QTc, in contrast to the findings of that study. There is one study in the literature suggesting that empagliflozin may prevent QTc prolongation induced by amitriptyline. However, this study only used a 10 mg dose [31]. Our study supports the idea that higher doses may produce more favourable results. Another study did not find a direct relationship between empagliflozin dose and cardiovascular effects, but suggested that increasing the dose of empagliflozin because of metabolic disorders associated with glycaemic control would not change cardiovascular effects. In other words, it argues that there is no need to increase the dose for cardiovascular effects and that if dose escalation is needed for metabolic reasons, it should be done accordingly [32]. Our study contributes to these considerations in the literature by emphasising that dose may be independently effective in preventing arrhythmias and by suggesting a new perspective on the need for dose adjustment.

Although the cardiovascular effects of SGLT-2 inhibitors were initially thought to be due to an increase in the amount of fasting ketones in the body, this was not clinically supported [33]. On the other hand, there are articles in the literature investigating how sodium-hydrogen exchanger-1 is inhibited, which is a potent receptor in the renal proximal tubule and myocyte membrane, thus keeping calcium, which causes myocardial toxicity, inside the cell [34, 35]. Another hypothesis is that renal medullary hypoxia is caused by SGLT-2 inhibitors due to increased active sodium reabsorption in the distal convoluted tubule, which leads to the formation of hypoxia-inducible factors, and this brings about the release of erythropoietin. The mass of erythrocytes increases, characterized by an increase in haematocrit, which enhances the provision of oxygen to the myocardium and decreases the mass of the left ventricle [36, 37]. In a review by Talha *et al*. [38], it was noted that many hypotheses have been put forward for the cardiovascular effects of SGLT-2 inhibitors, but it was argued that the exact mechanism of action is not yet clear. Unfortunately, we could not prove the results that would reveal the cardiac effects of SGLT-2 inhibitors, but we hope that it will be useful in future studies on this topic.

The QT, QTc, QTd, Tp-e and Tp-e/QTc values in our research were impaired in CAD and T2DM, as shown in similar studies. However, unlike previous studies, we investigated the response of these parameters to empagliflozin and found that the drug reduced them all. Although the mechanism of empagliflozin’s beneficial effects on the heart has not yet been elucidated, we hope that the results of our study will help to explain this mechanism. In particular, our finding that empagliflozin has a beneficial effect on the cardiac conduction system is a new contribution to the literature.

Very limited cases are studied in this research and there is a need for further publications with more cases. The observation period of the patients in this study was relatively limited. Therefore, multicenter studies with a longer follow-up are needed. In addition, because we did not include T2DM patients without CAD, we could not clarify whether the changes we observed in the ECG occurred in relation to T2DM or CAD, or only when both diseases coexisted. Finally, our study was not sufficient to clarify the mechanism of action of empagliflozin on the cardiovascular system.

## 5. Conclusions

In conclusion, our study sheds light on the possible advantages of empagliflozin in enhancing ventricular repolarisation factors in patients suffering from T2DM. We observed significant reductions in QTc, QTd, Tp-e and Tp-e/QTc after six months of empagliflozin treatment. These findings suggest that the use of empagliflozin is potentially linked to reduced incidence of ventricular arrhythmias. Further long-term studies need to be carried out to validate these effects and to investigate the underlying mechanisms. In addition to providing glycaemic control, empagliflozin may also have cardiovascular benefits, making it a valuable addition to the treatment options for people with T2DM who are at risk of cardiac complications.

## Data Availability

The datasets used in the current study are available from the corresponding author upon reasonable request.
